# High Serum Glial Fibrillary Acidic Protein and Low Serum Vitamin D Levels as Risk Factors for Cognitive Impairment in Ischemic Stroke Patients

**DOI:** 10.3390/neurolint18060120

**Published:** 2026-06-20

**Authors:** Patricia Patricia, Anak Agung Ayu Putri Laksmidewi, Kumara Tini, Anak Agung Ayu Meidiary, Ni Made Susilawathi, Ida Ayu Sri Wijayanti

**Affiliations:** 1 Neurology Resident, Faculty of Medicine, Udayana University, Prof. Dr. I.G.N.G. Ngoerah General Hospital, Bali 80113, Indonesia; patriciatian@gmail.com; 2 Department of Neurology, Faculty of Medicine, Udayana University, Prof. Dr. I.G.N.G. Ngoerah General Hospital, Bali 80113, Indonesia; kumara_tini@unud.ac.id (K.T.); meidiary@unud.ac.id (A.A.A.M.); susilawathi@unud.ac.id (N.M.S.); 3 Department of Neurology, Faculty of Medicine, Udayana University, Udayana University Hospital, Bali 80361, Indonesia; wijayanti@unud.ac.id

**Keywords:** ischemic stroke, GFAP, vitamin D, cognitive impairment, biomarker

## Abstract

Background: Cognitive impairment is a common complication after ischemic stroke and affects patients’ quality of life. Elevated glial fibrillary acidic protein (GFAP) and low vitamin D levels may contribute to neuroinflammation and impaired neuroplasticity, but their association with post-stroke cognitive impairment remains unclear. This study aimed to determine whether high serum GFAP and low vitamin D levels are risk factors for cognitive impairment in ischemic stroke patients. Methods: A prospective cohort study was conducted in patients with acute ischemic stroke. Serum GFAP and vitamin D levels were measured on the third day after stroke onset using an enzyme-linked immunosorbent assay (ELISA). Cognitive function was assessed two weeks after stroke onset using the Indonesian version of the Montreal Cognitive Assessment (MoCA-Ina). Data were analyzed using the chi-square test and multivariate logistic regression. Results: Seventy-six subjects were included in this study, of which 55 (72.4%) developed cognitive impairment. High serum GFAP (≥1.885 ng/mL) (RR = 1.755; 95% CI: 1.252–2.459; *p* = 0.001) and low vitamin D levels (<16.185 ng/mL) (RR = 1.773; 95% CI: 1.234–2.547; *p* = 0.001) were both associated with cognitive impairment. Multivariate analysis showed that high GFAP (AOR = 10.039; 95% CI: 2.484–40.569; *p* = 0.001) and low vitamin D levels (AOR = 6.640; 95% CI: 1.798–24.518; *p* = 0.005) were independent risk factors. Conclusions: Elevated serum GFAP and low vitamin D levels were independently associated with cognitive impairment after ischemic stroke and may serve as potential biomarkers for early risk stratification.

## 1. Introduction

Cognitive impairment imposes a heavy burden and is a major complication post-stroke, potentially diminishing a patient’s quality of life. It has been reported to affect up to 60% of stroke survivors within the first year after the cerebrovascular event [1,2,3]. Thus, there is an urgent need to identify reliable biomarkers to predict and stratify the risk of this cognitive decline.

Glial fibrillary acidic protein (GFAP) is a specific marker for astrocyte injury. Elevated GFAP levels reflect astrogliosis, microglial activation, and brain tissue injury, thus leading to a neuroinflammatory cascade [4,5]. Vitamin D also plays a role in the nervous system. Its active form and receptor are present in neurons and glial cells, and vitamin D signaling is involved in neurotrophic support, antioxidant defense, and immunomodulation [6,7]. Vitamin D has been found to play an essential indirect role in GFAP regulation; vitamin D deficiency upregulates NF-kB signaling, which can trigger astrogliosis and excessive GFAP expression [8,9]. Previous clinical studies have also reported that vitamin D deficiency may increase the overall incidence of cognitive impairment [10].

However, the association of these two biomarkers (GFAP and vitamin D) in assessing the specific risk of post-ischemic stroke cognitive impairment in this region has not been comprehensively studied. Therefore, this study was designed to determine whether elevated GFAP and low serum vitamin D levels serve as risk factors for post-stroke cognitive impairment.

## 2. Materials and Methods

### 2.1. Study Design and Participants

This prospective cohort study was conducted in the Neurology Department of Prof. Dr. I.G.N.G. Ngoerah Hospital, Denpasar. All patients diagnosed with acute ischemic stroke from December 2025 to February 2026 were identified. Patients were included if they met the criteria of being between 40 and 75 years old with a diagnosis of acute ischemic stroke within 72 h of onset confirmed by clinical signs, symptoms, and head CT non-contrast or angiography; good consciousness and pre-stroke cognitive function (short IQ code < 3.48); and mild to moderate stroke severity based on NIHSS. Patients were excluded if they met the criteria of having a history of previous stroke; language disorders/aphasia; depression (HDRS score > 13); traumatic brain injuries or other intracranial diseases such as central nervous system (CNS) infections, brain tumors, seizures or epilepsy, dementia, or Parkinson’s disease; chronic systemic disease, such as kidney or chronic liver disease, HIV infection, and autoimmune diseases; alcohol consumption; intestinal malabsorption syndrome; or vitamin D supplement use within the past month. Patients were also excluded if they were vegetarian, had severe visual and hearing impairments, were unable to read and write due to illiteracy, or refused to participate.

### 2.2. Data Collection

Data collection included all patients who participated in this study. Demographic and clinical data were collected, including age, gender, level of education, nutritional status, GFAP and vitamin D levels, type of stroke, hypertension, diabetes, cardiac disorder, and NIHSS. Neuroimaging, such as computed tomography (CT/CTA) angiography of the brain, was conducted for all patients at the time of admission. The Montreal Cognitive Assessment questionnaire, Indonesian version (MoCA-Ina), was administered to determine cognitive impairment. It is a brief, validated, and easy-to-use tool for identifying cognitive impairment with good sensitivity and specificity, and scores < 26 indicate such impairment. The Hamilton Depression Rating Scale was applied to determine the presence of depression (score ≥ 8).

All participants then underwent venous blood sampling conducted by experts for the laboratory examination of serum GFAP and vitamin D (25(OH)D3) levels measured in ng/mL, which were collected after 3 days of ischemic stroke onset using an enzyme-linked immunosorbent assay (ELISA) in the Clinical Pathology Laboratory, Medical Faculty of Universitas Udayana. Blood sampling was standardized on the third day after stroke onset to minimize variability in biomarker collection timing among participants and ensure that measurements were obtained during the acute phase of ischemic stroke. Serum GFAP concentrations were measured using a commercially available ELISA kit (Bioassay Technology Laboratorium, Shanghai, China; Catalog No. E2094Hu), as were those of serum 25-hydroxyvitamin D3 [25(OH)D3] using another commercially available ELISA kit (Elabscience, Wuhan, China; Catalog No. E-EL-0015). Patients were then followed up and, at 2 weeks, were examined for their cognitive function. The study protocol was approved by the local ethics committee of Prof. Dr. I.G.N.G. Ngoerah hospital, and informed written consent was obtained from all patients or their relatives to participate in this study, with approval code 2718/UN14.2.2.VII.14/LT/2025.

### 2.3. Statistical Analysis

We used SPSS version 27 (Statistical Package for the Social Sciences) to analyze all the collected data. Categorical variables are presented as frequencies and were compared using the chi-square and Fisher’s exact tests when appropriate. Quantitative variables are described as the median and range or means and standard deviations, according to data type. All the analyzed variables are presented as nominal ones. The cut-off for high GFAP and low vitamin D serum levels was obtained through the receiver operating characteristic (ROC). ROC curve analysis was performed to establish appropriate cut-off values and robustly evaluate the sensitivity and specificity of the investigated indicators for risk factors. All variables with a significance value less than 0.25 in the bivariate analysis were included in the multivariate analysis. Initial analysis was performed using a modified Poisson regression with robust variance estimation to obtain relative risk estimates. Before finalizing the model, we examined the Poisson model’s diagnostics. The diagnostic evaluation showed a deviance/df ratio of 0.4 and a Pearson χ^2^/df ratio of 0.27, thus indicating severe underdispersion. Poisson regression is not maintained under such conditions, as it carries a significant risk of overestimating the standard error and weakening the statistical power of the test; therefore, subsequent multivariate analysis used logistic regression. A *p*-value < 0.05 was regarded as statistically significant.

## 3. Results

**Figure 1:**
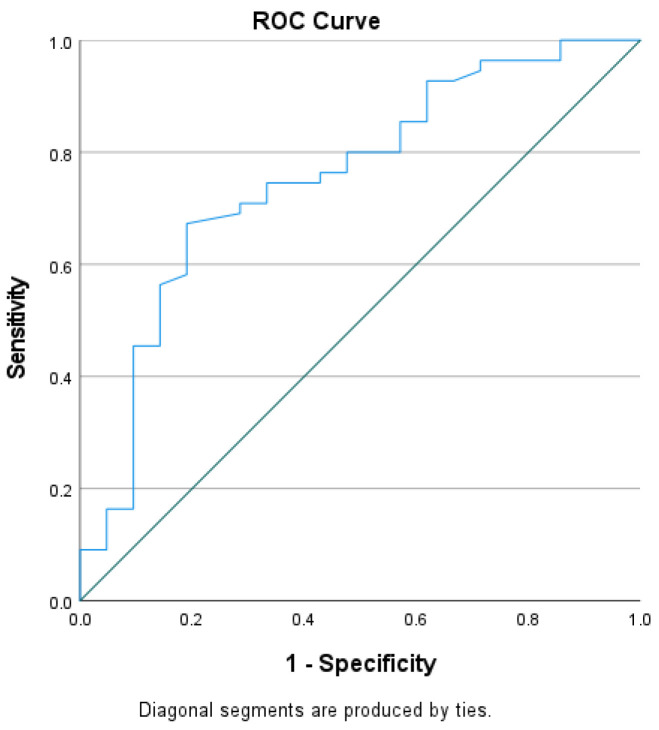
The ROC curve of the GFAP level and cognitive impairment in acute ischemic stroke patients. The AUC based on this curve is 75.3%. ROC, receiver operating characteristic; AUC, area under the curve.

**Figure 2:**
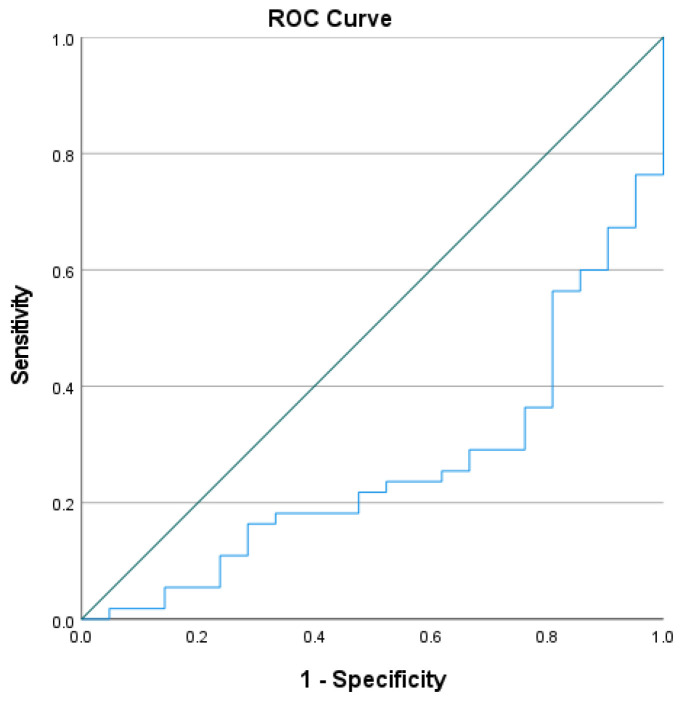
The ROC curve of the vitamin D level and cognitive impairment in acute ischemic stroke patients. The inverse AUC based on this curve is 26%. ROC, receiver operating characteristic; AUC, area under the curve.

A total of 76 patients were eligible for this study, but 4 were lost to follow-up. Cognitive impairment was identified in 55 patients (72.37%) (Table 1). The ROC method yielded an area under the curve (AUC) value of 75.3% [95% confidence interval (CI) 62.8 to 87.8%] for GFAP (Figure 1). A GFAP cut-off value of 1.885 ng/mL resulted in 67.3% sensitivity and 81% specificity (Figure 1). In terms of vitamin D, the ROC method yielded an inverse area under the curve (AUC) value of 26% [95% confidence interval (CI) 14.1 to 37.8%] (Figure 1). A vitamin D cut-off value of 16.185 ng/mL resulted in a sensitivity of 70.9% and specificity of 76.2% (Figure 2). Ischemic stroke patients with high GFAP serum levels had a significantly higher risk of experiencing cognitive impairment compared to those with normal cognitive function (RR 1.755; 95% CI [1.252–2.459]; *p* = 0.001) (Table 2).

Moreover, ischemic stroke patients with lower vitamin D levels had a significantly higher risk of experiencing cognitive impairment compared to those with normal cognitive function (RR 1.773; 95% CI [1.234–2.547]; *p* = 0.001) (Table 3). There were no other factors associated with cognitive impairment in this research (Table 4). The variables GFAP, vitamin D, and type of stroke had significance values less than 0.25, so they were also assessed in multivariate analysis. In the multivariable logistic regression analysis, higher GFAP (AOR 10.039; 95% CI [2.484–40.569]; *p* = 0.001) and low vitamin D levels (AOR 6.640; 95% CI [1.798–24.518]; *p* = 0.005) were associated with cognitive impairment (Table 5).

## 4. Discussion

In this prospective cohort study, we demonstrated that post-stroke cognitive impairment (PSCI) occurred in a substantial proportion of patients with acute ischemic stroke, at 72.37%. Baseline characteristics showed that male patients were more prevalent, which is consistent with previous studies reporting a higher incidence of stroke in men, particularly in the 45–74 age group, which is possibly due to the protective vascular effects of estrogen in women [11]. The educational level was relatively balanced in this study; however, higher education is known to be associated with greater cognitive reserve, thus enabling better compensation after brain injury through more efficient neural network recruitment [12,13,14]. Nutritional status was predominantly normal, which is in line with previous studies; however, it remains relevant because it is associated with systemic inflammation and endothelial dysfunction, both of which may influence stroke outcomes [15,16]. Most patients had small vessel occlusion (SVO), which is consistent with prior epidemiological data and reflects underlying small vessel pathology such as lipohyalinosis leading to subcortical hypoperfusion [17,18]. Most patients had moderate stroke severity, which may also contribute to the relatively high prevalence of early cognitive impairment. Obesity was considered a potential confounder because adiposity has been linked to chronic systemic inflammation, central neuroinflammation, cognitive dysfunction, and lower circulating 25(OH)D levels. Therefore, adjusting for obesity helped to reduce residual confounding in the association between vitamin D status and post-stroke cognitive impairment [19,20,21].

The association between elevated GFAP levels and PSCI is biologically plausible. GFAP is an astrocyte-specific intermediate filament protein whose levels increase following astrocyte activation and ischemic injury. Elevated GFAP levels reflect astroglial damage, blood–brain barrier disruption, and the extent of brain injury [22]. Previous studies have shown that higher serum GFAP levels are associated with worse cognitive outcomes and greater white matter damage after stroke [23]. Reactive astrocytes release proinflammatory cytokines that exacerbate neuroinflammation and contribute to neuronal dysfunction. Experimental studies have also demonstrated that increased GFAP expression in the hippocampus is associated with memory impairment, while the inhibition of astrocyte activation improves synaptic function [24].

Vitamin D deficiency was also significantly associated with PSCI in this study. Vitamin D plays a crucial role in neuroprotection by modulating inflammation, oxidative stress, and neuronal survival [6]. Low 25(OH)D3 levels have been associated with poorer cognitive performance, larger infarct size, and worse functional outcomes after stroke [25,26]. Mechanistically, vitamin D deficiency promotes a proinflammatory microglial phenotype, increases blood–brain barrier permeability, and impairs endothelial function, thus leading to ongoing neuronal injury [27]. In addition, vitamin D regulates neurotrophic factors such as IGF-1, which are essential for neuronal repair and synaptic plasticity [28].

The combined presence of elevated GFAP levels and low vitamin D likely reflects two complementary pathological processes: structural brain injury and reduced neuroprotective capacity. Patients with both abnormalities may therefore be more vulnerable to PSCI, which may explain the independent associations observed in this study. From a clinical perspective, PSCI is associated with poorer functional outcomes and an increased risk of recurrent stroke and mortality. The early identification of high-risk patients is therefore essential. While cognitive screening tools remain important, biomarkers such as GFAP and vitamin D may provide additional value, particularly in the acute phase when neurological deficits limit cognitive assessment.

This study has several strengths. First, its prospective design allows for temporal assessment between biomarker levels and cognitive outcomes. Second, biomarkers were measured in the acute phase, which is clinically relevant for early risk prediction. Third, multivariable analysis was performed to adjust for potential confounding factors, thus strengthening the validity of the observed associations. However, several limitations should be acknowledged. First, the sample size was relatively small and derived from a single center, which may limit generalizability. Biomarker measurements were obtained at a single time point, which may not capture dynamic changes over time. Third, acute inflammatory and metabolic responses after stroke, as well as intra-individual variability in vitamin D status, may have influenced measured vitamin D concentrations. Fourth, frailty, sarcopenia, physical activity, sunlight exposure, and nutritional factors were not formally evaluated and may have acted as residual confounders. Fifth, lesion volume and location, hippocampal involvement, and cerebral perfusion parameters were not systematically analyzed, thus preventing the assessment of their contribution to cognitive outcomes. Finally, the absence of a healthy control group limits conclusions regarding the specificity of these associations to ischemic stroke populations. Future studies with larger multicenter cohorts, repeated biomarker measurements, and longer follow-up periods are needed to validate these findings and explore causal relationships. Furthermore, interventional studies investigating whether the correction of vitamin D deficiency or modulation of astrocytic injury can improve cognitive outcomes would provide valuable insights into potential therapeutic strategies.

## 5. Conclusions

High serum GFAP (≥1.885 ng/mL) and low serum vitamin D levels (<16.185 ng/mL) measured during the acute phase of ischemic stroke were independently associated with early post-stroke cognitive impairment. These biomarkers may provide additional value for identifying patients at increased risk of cognitive dysfunction following stroke. However, given the observational nature of this study and the potential influence of unmeasured confounding factors, further large-scale multicenter studies with longer follow-up periods are required to validate these findings and clarify their causal relationship with post-stroke cognitive outcomes.

## Figures and Tables

**Table 1:** Demographic and clinical characteristics of the two groups.

Variables	All Subjects (*n* = 76)	Cognitive Impairment (*n* = 55)	No Cognitive Impairment (*n* = 21)
Age (mean ± SD) years	54.39 ± 8.095	54.47 ± 8.395	54.19 ± 7.441
Sex, *n* (%)			
Male	47 (61.8%)	35 (63.6%)	12 (57.1%)
Female	29 (38.2%)	20 (36.4%)	9 (42.9%)
Level of education, *n* (%)			
Low (≤9 years)	32 (42.1%)	22 (40%)	10 (47.6%)
High (>9 years)	44 (57.9%)	33 (60%)	11 (52.4%)
Nutritional status, *n* (%)			
Normal	44 (57.9%)	31 (56.4%)	13 (61.9%)
Underweight	1 (1.3%)	0 (0%)	1 (4.8%)
Overweight	14 (18.4%)	10 (18.2%)	4 (19%)
Obesity	17 (22.4%)	14 (25.5%)	3 (14.3%)
GFAP serum levels, (median [min-max]), ng/mL	1.98 (0.17–19.64)	2.23 (0.36–19.64)	1.58 (0.17–13.27)
Vitamin D serum levels, (median [min-max]), ng/mL	14.12 (1.14–264.86)	10.72 (1.14–231.08)	21.31 (5.67–264.86)
Type of strokes, *n* (%)			
Small vessel occlusion	61 (80.3%)	41 (74.5%)	20 (95.2%)
Large artery atherosclerosis	11 (14.5%)	10 (18.2%)	1 (4.8%)
Cardioembolic	4 (5.3%)	4 (7.3%)	0 (0%)
Hypertension *n* (%)			
Yes	37 (48.7%)	28 (50.9%)	9 (42.9%)
No	39 (51.3%)	27 (49%)	12 (57.1%)
Type 2 DM, *n* (%)			
Yes	30 (39.5%)	23 (29.1%)	7 (33.3%)
No	46 (60.5%)	32 (70.9%)	14 (66.7%)
Cardiac disease, *n* (%)			
Yes	10 (13.2%)	10 (18.2%)	0 (0%)
No	66 (86.8%)	45 (81.8%)	21 (100%)
NIHSS, *n* (%)			
Mild (1–4)	27 (35.5%)	18 (32.7%)	9 (42.9%)
Moderate (5–15)	49 (64.5%)	37 (67.3%)	12 (57.1%)

**Table 2:** Bivariate analysis of serum GFAP levels and cognitive impairment in ischemic stroke patients.

Variables		Cognitive Impairment		RR	*p*
		Yes *n* (%)	No *n* (%)	(CI 95%)	
GFAP (ng/mL)	High (≥1885 ng/mL)	37 (90.2%)	4 (9.8%)	1.755 (1.252–2.459)	0.001 *
	Low (<1885 ng/mL)	18 (51.4%)	17 (48.6%)		

*: statistically significant; RR: relative risk; CI: confidence interval.

**Table 3:** Bivariate analysis of serum vitamin D levels and cognitive impairment in ischemic stroke patients.

Variables		Cognitive Impairment		RR	*p*
		Yes *n* (%)	No *n* (%)	(CI 95%)	
Vitamin D (ng/mL)	Low (<16,185 ng/mL)	39 (88.6%)	5 (11.4%)	1.773 (1.234–2.547)	0.001 *
	Not Low (≥16,185 ng/mL)	16 (50%)	16 (50%)		

*: statistically significant; RR: relative risk; CI: confidence interval.

**Table 4:** Bivariate analysis of covariate variables and cognitive impairment in ischemic stroke patients.

Variables	Cognitive Impairment		RR (95% CI)	*p*
	Yes *n* (%)	No *n* (%)		
Sex			1.080 (0.803–1.452)	
Male	35 (74.5%)	12 (25.5%)		0.602 ^a^
Female	20 (69%)	9 (31%)		
Levels of education				
Low (≤9 years)	22 (68.8%)	10 (31.3%)	0.917	0.547 ^a^
High (>9 years)	33 (75%)	11 (25%)	(0.686–1.224)	
Nutritional status				
Obesity	14 (82.4%)	3 (17.6%)	1.185	0.369 ^b^
Not obesity	41 (69.5%)	18 (30.5%)	(0.898–1.564)	
Type of stroke				
Small vessel occlusion	41 (67.2%)	20 (32.8%)	0.720	0.054 ^b^
LAA + cardioembolic	14 (93.3%)	1 (6.7%)	(0.577–0.899)	
NIHSS				
Mild	18 (66.7%)	9 (33.3%)	0.883	0.409 ^a^
Moderate	37 (75.5%)	12 (24.5%)	(0.647–1.205)	
Hypertension				
Yes	28 (75.7%)	9 (24.3%)	1.093	0.53 ^a^
No	27 (69.2%)	12 (30.8%)	(0.828–1.443)	
Type 2 DM				
Yes	23 (76.7%)	7 (23.3%)	1.102	0.499 ^a^
No	32 (69.6%)	14 (30.4%)	(0.837–1.451)	
Cardiac disease				
Yes	10 (100%)	0 (0%)	1.467	0.054 ^b^
No	45 (68.2%)	21 (31.8%)	(1.244–1.729)	

^a^ Chi-square test; ^b^ Fisher’s exact test.

**Table 5:** Multivariate logistic regression analysis.

(a) Before obesity variable inserted.			
Characteristics	AOR	95% CI	*p*
Step 1			
High serum levels of GFAP	5.839	1.564–21.799	0.009 *
Low serum levels of vitamin D	5.110	1.315–19.862	0.019 *
Type of stroke	0.816	0.07–9.461	0.871
Step 2			
High serum levels of GFAP	5.830	1.562–21.756	0.009 *
Low serum levels of vitamin D	5.340	1.515–18.825	0.009 *
(b) After obesity variable inserted.			
Characteristics	AOR	95% CI	*p*
Step 1			
High serum levels of GFAP	9.776	2.418–39.519	0.001 *
Low serum levels of vitamin D	5.721	1.360–24.068	0.017 *
Obesity	4.692	0.879–25.035	0.070
Type of stroke	0.582	0.051–6.624	0.663
Step 2			
High serum levels of GFAP	10.039	2.484–40.569	0.001 *
Low serum levels of vitamin D	6.640	1.798–24.518	0.005 *
Obesity	4.922	0.938–25.830	0.060

*: statistically significant; AOR: adjusted odds ratio; CI: confidence interval.

## Data Availability

The original contributions presented in this study are included in the article. Further inquiries can be directed to the corresponding author.

## Abbreviations

AOR	Adjusted odds ratio
AUC	Area under the curve
CI	Confidence interval
CNS	Central nervous system
CTA	Computed tomography angiography
ELISA	Enzyme-linked immunosorbent assay
GFAP	Glial fibrillary acidic protein
HDRS	Hamilton Depression Rating Scale
HIV	Human immunodeficiency virus
IGF-1	Insulin-like growth factor 1
IQ Code	Informant Questionnaire for Cognitive Decline in the Elderly
LAA	Large artery atherosclerosis
MoCA-Ina	Indonesian version of the Montreal Cognitive Assessment
NF-kB	Nuclear factor kappa-light-chain-enhancer of activated B cells
NIHSS	National Institutes of Health Stroke Scale
PSCI	Post-stroke cognitive impairment
ROC	Receiver operating curve
RR	Relative risk
SVO	Small vessel occlusion
